# Analysis of Water, Ethanol, and Fructose Mixtures Using Nondestructive Resonant Spectroscopy of Mechanical Vibrations and a Grouping Genetic Algorithm

**DOI:** 10.3390/s18082695

**Published:** 2018-08-16

**Authors:** Pilar García Díaz, Juan Antonio Martínez Rojas, Manuel Utrilla Manso, Leticia Monasterio Expósito

**Affiliations:** Department of Signal Theory and Communications, University of Alcalá, Polytechnic School, 28871 Alcalá de Henares, Madrid, Spain; juanan.martinez@uah.es (J.A.M.R.); manuel.utrilla@uah.es (M.U.M.); leticia.monasterio@edu.uah.es (L.M.E.)

**Keywords:** haptic sensors, wine chemistry, nondestructive analysis, feature selection, extreme learning machine, classification

## Abstract

A new haptic sensor that is based on vibration produced by mechanical excitation from a clock coupled to a resonant cavity is presented. This sensor is intended to determine the chemical composition of liquid mixtures in a completely non-destructive method. In this case, a set of 23 samples of water, ethanol, and fructose mixtures has been used to simulate different kinds of alcoholic beverage. The spectral information from the vibrational absorption bands of liquid samples is analyzed by a Grouping Genetic Algorithm. An Extreme Learning Machine implements the fitness function that is able to classify the mixtures according to the concentration of ethanol and fructose. The 23 samples range from 0%–13% by volume of ethanol and from 0–3 g/L of fructose, all of them with different concentration. The new technique achieves an average classification accuracy of 96%.

## 1. Introduction

Alcoholic beverages, like wine and beer, are important products in many countries with a considerable economic impact in the industry. The final product quality must be ensured for which many analytical methods have been researched. Some beverages are internationally recognized by their qualities, thus special protection is applied to avoid fraud. This demands increasingly sensitive, cheap, fast, and accurate non-destructive sensors for characterization and verification. As can be seen in [1,2,3,4], the most used non-destructive analytical technique is vibrational spectroscopy (near-infrared, mid-infrared, and Raman), although other methods are also applied, like electronic tongues [5,6] and nuclear magnetic resonance (NMR) [7,8].

Haptic sensors using pressure (firmness testers) have been used from some time in food science and technology [9,10]. Obviously, these systems are not suitable for liquid analysis and rigid container non-destructive analysis. However, a system that is based on the mechanical vibrational excitation of liquid samples can circumvent the limitations of pressure based tactile sensors. Although stress and surface waves have been used previously for nondestructive testing of wood [11] and watermelons [12,13,14], as far as we know, this is the first time that such a sensing approach for the chemical composition study of liquid foods is proposed.

Other acoustic techniques work generally in the frequency range of 1–100 MHz [15], but this one analyzes audible sounds that are produced by ticking vibrations in order to improve the penetrating properties of the sensor, thus avoiding the large attenuation of high frequency ultrasounds.

A more direct approach to the determination of physicochemical properties of fruit can be seen in [16]. They obtain the properties of fruit by correlation between the results of direct destructive determination of its chemical components and previous measurements of ultrasonic response of the same fruit. However, in acoustic spectroscopy, the measured sound parameters are attenuation and sound velocity, instead of the spectral response of the material. The use of sound that is generated by very low energy vibrations presents many advantages. It is non-destructive, can be used with intact containers, and it is penetrating enough to explore large volumes of liquid. The analysis of the spectral characteristics of each sample can be made with powerful free or commercial software. Finally, no special coupling or immersed probes are necessary as in the case of ultrasound testing.

The main innovation of this work is the proof that acoustic spectroscopy of liquids, placed the native container with unbroken seal, combined with powerful data mining algorithms, can provide the composition of ternary water-fructose-ethanol mixtures while using a very simple and cost effective experimental setup. Audible sound is able to penetrate most sample containers, so that some limitations of other methods, mainly optical ones, are eliminated. The spectral analysis of resonant mechanical vibrations can be used to study the chemical composition of liquid foods, and not only the mechanical properties of solid foods, like ripeness or texture.

## 2. Material and Methods

A new haptic sensing technique is presented in this work bioinspired by vibrational touch performance related with certain resonances. This new method performs a direct study of the spectral signatures of vibrational resonances inside a cavity in order to detect the chemical substances in liquids. The experimental system was composed by a mechanical clock as a ticking source, and an isolating platform made from polyethylene foam, a piezoelectric microphone with flat response between 20 Hz and 20 kHz, a resonating cavity made from polypropylene, and the fluid sample. Spectral information in the audible sound frequency range, as obtained from the vibrational absorption bands of the liquid sample inside a reference container, is analyzed by a heuristic classification algorithm. The liquid sample is placed on the surface of cylindrical box that is acting like a mechanical resonator excited by a stable ticking source from a mechanical clock. In an abstract sense, this bioinspired vibrational method can be seen as a cheap and powerful alternative to the previously cited optical and nuclear resonance techniques.

**Figure 1:**
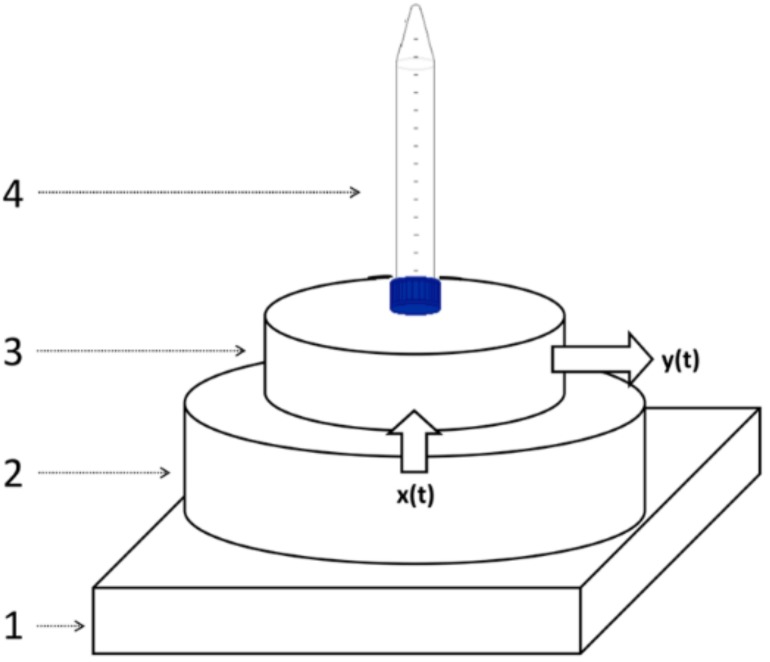
Schematic diagram of the experimental setup. From the bottom up there is a polyethylene platform (1) to reduce the influence on the acoustic output; (2) a ticking generator source of the input signal *x(t)*; a resonator (3) and the fluid sample (4) for classifying through the output signal *y(t)*.

Figure 1 shows the schematic diagram of the experimental setup. The element number 1 represents the polyethylene platform which reduces the influence of the contact support in the acoustic output picked up by the microphone. Box 2 plots the ticking source which generates the input signal *x(t)* on the resonating cavity. This cavity has a diameter of 15 cm. Box 3 depicts the resonating cavity which includes the piezoelectric microphone that picks up the output *y(t)*. The fluid sample (element 4 in the Figure 1) was placed on the upper surface of the resonator 3. Each audio recording had a duration of 30 s, which was a very good compromise between accuracy, precision and measuring time. This recording time was long enough to eliminate the errors due to the fluctuation of the ticking source from one pulse to another (changes among different spectra from the same sample were below 1%), but short enough to make this technique practical. Obviously, the longer the recording time, the better the spectral precision due to fluctuation averaging.

The microphone was connected to a PC sound card. The measurements were taken with a recording rate of 44,100 Hz by means of the Praat software [17].

The experiments have been carried out with a set of 23 samples of water solutions with different concentrations of ethanol and fructose. The volume of each sample was 50 mL. Distilled water was used as solvent. Pharmaceutic grade 96% ethanol and food grade pure fructose (>99%) were used for the liquid samples.

The concentrations of ethanol and fructose were:

- Ethanol: 0, 6, 10, 11, 12, and 13% by volume.
- Fructose: 0, 1, 2, and 3 g/L.

For ethanol volume measurement, polypropylene beakers that are compliant with the ISO 7056 norm were used. In case of real drinks, we would use the native container. We decided to use the polypropylene beakers because the water solutions were prepared as simulated beverages for the experiment. The beakers were new and sterile when used, so no pretreatment or washing procedure was applied. The beakers had a screw cap of plastic. The beaker volume was 50 mL with marks every 10 mL. For finer volume determination, new and sterile medical plastic syringes were used.

The fructose mass for the samples were determined by means of an analytical balance (Ohaus Adventurer, OHAUS Corporation, Parsippany, NJ, USA) with a readability of 0.01 g.

First, samples of ethanol and water were prepared and then fructose was added. The samples were sealed in identical new sterile volume marked polypropylene tubes with polyethylene screw caps. Samples were numbered as the Table 1 shows and they were kept in a box for a week before measurements were performed to ensure that complete dilution was achieved, and no bubbles were found inside.

The 23 samples were numbered with numeric labels from sample 2 to sample 24. The Table 1 registers the composition of each sample. The numerical label 1 would correspond to the sample that was composed by pure water. Because we want to emulate alcoholic beverages and substitutes, we have not included the plain water in the experiments.

All of the measurements were done during the same day with careful manipulation of the samples in order to avoid the formation of bubbles or wall drops. Each measurement takes 30 s and they were taken in a consecutive way. The clock ticking sounds were monitored in order to assure their precision by means of the Tickoprint Android App. No anechoic chamber was used for the recordings, but a quiet laboratory room. The noise level was measured with the same system without samples at regular intervals in order to ensure that all sound recordings were made in the same conditions. The acoustical response of samples to the resonant ticking sounds was recorded by means of the Praat software program during 30 s with a sampling rate of 44,100 kHz.

Each measurement was fragmented in different 6 s intervals ([1–6], [2–7], [3–8], …, [24–29]). The input data of the classification algorithm are the spectra of the audio measurements of 6 s in duration. The experiment was carried out with 24 audio measurements of each of the 23 samples of different composition. That means a total of 24 × 23 = 552 audio measurements. The power spectrum of every interval was made using the default Praat options. The power spectrum (or power spectral density) is calculated as:

(1) $$\;S_{xx}\left(\omega \right)=\underset{T\to \infty }{\lim}Ε\left[{\left|\hat{x}\left(\omega \right)\right|}^{2}\right]\;$$

where,

(2) $$\begin{array}{ll} \;Ε\left[{\left|\hat{x}\left(\omega \right)\right|}^{2}\right] & =Ε\left[\frac{1}{T}\int_{0}^{T}x^{*}\left(t\right)e^{i\omega t}dt\int_{0}^{T}x\left(t\prime \right)e^{-i\omega t^{\prime }}dt\prime \right]\\ & =\frac{1}{T}\iint_{0}^{T}Ε\left[x^{*}\left(t\right)x\left(t\prime \right)\right]e^{i\omega \left(t-t^{\prime }\right)}dtdt\prime \; \end{array}$$

In the discrete case, a single approximation is:

(3) $${\tilde{S}}_{xx}\left(\omega \right)=\frac{{\left(\Delta t\right)}^{2}}{T}{\left|\sum_{n=1}^{N}x_{n}e^{-i\omega n\Delta t}\right|}^{2},$$

considering a finite window of 1⩽n⩽N and a signal sampled at discrete times $x_{n}=x\left(n\Delta t\right)$ for a total measurement period T=NΔt [18].

Then, a cepstral smoothing of 100 Hz and a decimation procedure were applied in order to reduce the number of points to a reasonable size (1310 points) without losing the main peak structure of the spectra.

The cepstrum is the result of taking the inverse Fourier transform (IFT) of the logarithm of the estimated spectrum of a signal. Generally, the power cepstrum is used in human speech applications, which is defined as:

(4) $${\left|{ℱ}^{-1}\left\{\log\left({\left|ℱ\left\{f\left(t\right)\right\}\right|}^{2}\right)\right\}\right|}^{2}.$$

The cepstral smoothing algorithm averages the spectrum data while using the cepstral results and selecting a frequency interval in Hz [19].

In summary, the classification algorithm will process 552 input data, each of them being a spectrum that is defined by 1310 values in the frequency range 20 Hz–22.05 kHz.

## 3. Algorithm for Clustering Problem

The spectral information from the vibrational absorption bands of liquid samples is analyzed by the Grouping Genetic Algorithm (GGA) in order to determine the chemical composition of the liquid mixtures in a completely non-destructive method. The algorithm performs a classification according to the spectral response to the vibrational stimulation from the ticking source. The GGA is a modified Genetic Algorithm (GA) for solving clustering and grouping problems. This section provides a brief overview of GA and GGA.

### 3.1. The Genetic Algorithm as Evolutionary Algorithm

The Genetic Algorithm belongs to the evolutionary optimization algorithms category. Optimization techniques are generally applied to solve problems where it is not possible to find the optimal solution due to the existence of opposing criteria. It is extremely difficult to obtain the optimal solution and, in general, we do not know if there is a single optimal solution, several solutions, and how many solutions are close enough to the optimum, being that these are much easier to find than the optimal one. The objective is to find a suitable solution, enough nearby to the optimal one, with a limited execution time. We are not interested to find exactly the optimal solution after unacceptable computing time. The characteristic 'evolutionary' means that the algorithm analyzes and processes solutions across several stages, so solutions go through an evolutionary process from the first stage to the last one.

The GA is bio-inspired in the Charles Darwin theory or theory of evolution by natural selection. A population of individuals fighting for survival corresponds to a set of solutions of the optimization problem. Individuals with better fitness will be survive with higher probability than others having worse aptitudes. So, the survival of individuals depends on their fitness and chance in life. The passing of time makes the species to conform to the environment, as the GA will find better solutions of the optimization problem after several generations. The fitness function that is used in this paper to carry out the classification of spectra data is the Extreme Learning Machine (ELM). We discuss the ELM in the following section of the paper.

**Figure 2:**
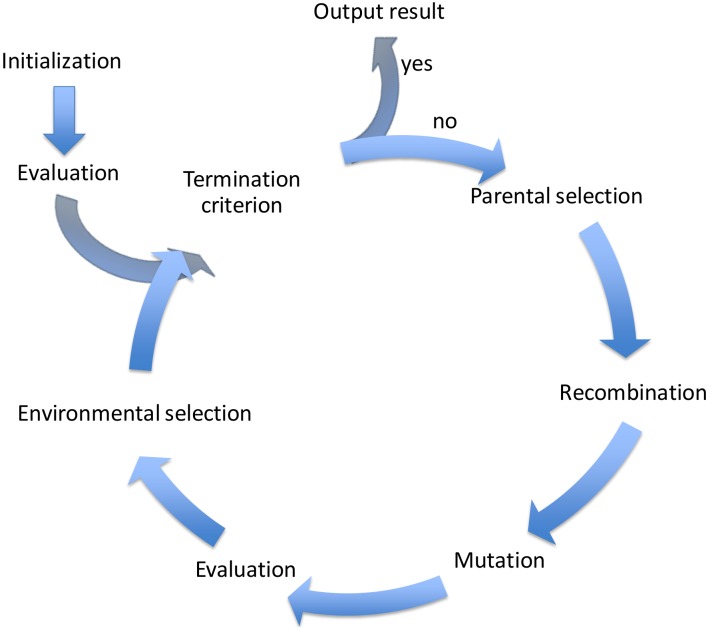
General flow chart of the Genetic Algorithm. A population of coded solutions progresses and is transformed during a finite number of consecutive generations.

The Figure 2 represents the general flow chart of the GA, where a complete cycle depicts a generation of the evolutionary process. Each one executes operations of: fitness computing, selection, recombination or crossover, and mutation. The process starts with an initial population composed of random solutions. Each solution is characterized by a fitness value, which is a measurement/degree of how well it solves the optimization problem: a solution with better fitness value means that it is closer to the unknown optimal solution than others with weaker fitness value. Due to the initial solutions being randomly created, most of them are sure very far from any optimal solution. The algorithm evaluates each solution of the initial population in order to register their fitness values.

After the initial evaluation, the algorithm generates new solutions as offspring the current population. The method to create a new solution is usually based on the contents of two existing solutions, inspired on the creation of offspring from the genetic makeup of their parents in real live. The GA adjustment to solve a particular problem determines the specific rules of parental selection and recombination with a consistent pattern in the encoding solution.

As Darwinian Theory, there is a small probability of unexpected mutation. The GA models the mutation as a random modification in a piece of the solution code. After recombination and mutation, the fitness value of the offspring is calculated. Then, the offspring is added to the current population, therefore the population size increases to living together in an environment with restricted resources. The environmental selection controls the population size discarding some solutions. There are different discard methods, GA usually applies tournaments. Solutions with better fitness will generally win the tournaments, but it also depends on chance.

A new generation starts, and the execution comes back to the parental selection for recombination and following operations. The process finished when some stop conditions matches. Stop conditions for GA usually consist of a maximum number of generations or reaching the convergence of population.

### 3.2. The Grouping Genetic Algorithm

The GGA is a modified genetic algorithm for solving clustering and grouping problems [20,21,22,23]. The expression ’grouping’ refers to a technique that takes advantage of special encoding strategies and searching operators in order to obtain compact hierarchical arrangements in grouping-based problems with a high performance in terms of a hierarchy-dependent metric [24].

The solution coding of a GGA has two different sections: ‘assignment part’ and ‘grouping part’. Both sections are arrays of elements. The information about the classification is not only inside the content of array, also in the length of the arrays. The length of the assignment part is the size of elements to classify. The length of the grouping part matches with the number of groups. Note that the length of the solutions is variable, because each solution could consider different number of groups.

**Figure 3:**
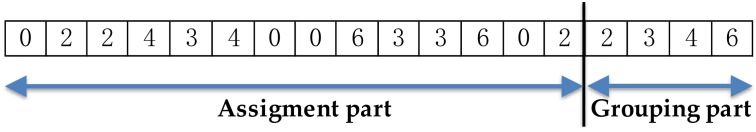
Example of coding a solution for the algorithm for the Grouping Genetic Algorithm (GGA). The assignment part has the length of the set of items to be grouped (14 elements in the example). The grouping part identifies the existing groups (groups 2, 3, 4, and 6 in the example). Each element of the assignment part is related to a single group by storing the group identifier. All the elements related to the same group contain the same group identifier. The value 0 means that the element is not yet associated with any group.

The Figure 3 shows a simple example of solution coding. This individual has four groups (length of the grouping part is four), named 2, 3, 4, and 6 (content of the array). It is not required using consecutive numbers to identify categories, neither starting at 1. The content of the assignment part array is the group or type which is associated each element. If the element is not yet classified, the array stores a zero value in the array. In this way, the example in Figure 3 indicates that the first element is not yet associated to any group, whereas the second, third, and last elements of the array are assigned to group 2, the forth is typed as class 4, and so on.

The code of a solution is formed by two parts: assignment part and grouping part. The grouping part collects the different groups of the solution. The value that is stored in the assignment array represents the group that is classified the corresponding element of the array.

The GGA keeps the general flow process described in Figure 2 for GA because GGA is a modification of this. However, the recombination and mutation operations have different characteristics than GA. The mutation of an individual can be understood as a recombination with a random individual, so we explain more in detail only the crossover operator.

Figure 3 represents an example of recombination of individual A and B in the GGA. For clear explanation, the coding of all cases emphasizes the assignment and grouping parts with a vertical line separating the arrays.

**Figure 4:**
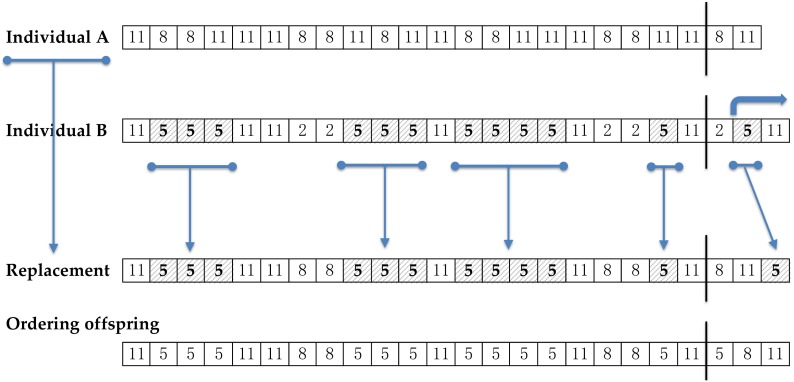
Example of recombination of individuals A and B by a single crossing point in the grouping part of B. The generated individual is based on the composition of A, on which are added the new groups that appear to the right of the crossing point in B. The groups selected by the crossing point are 5 and 11. Group 11 exists already in A, then only the group number 5 must be added. The assignment part of the resulting individual is modified according to the elements associated to group number 5 in B.

The recombination operation begins taking a copy of individual A as the offspring. Then, a fragment of the grouping part of the individual B is randomly selected. The example in the Figure 4 considers the sub-array [15,17] of individual B. Only the new content of this sub-array is added over the offspring. The example gets the group number 5 because the class 11 is already in the copy. The next step consists of copying to the offspring the complete sets of elements in the individual B associated to the added groups. In Figure 4, all elements of the individual B belonging to the class number 5 are updated in the offspring. Finally, the grouping part could be re-written in ascending order.

The new offspring is a modified copy of the predecessor A. This copy is added some new groups of the individual B and reassigned the elements belonging to the new groups, like the predecessor B.

### 3.3. The Fitness Function: The Extreme Learning Machine

An effective fitness function of the GGA presents two major properties: the regressor is as accurate as possible, and the evaluation process is as fast as possible in order to not exceed the total processing time of the algorithm. The Extreme Learning Machine (ELM) matches both requirements: it is a simple machine learning algorithm that achieves a good generalization performance with extremely fast speed [25]. ELM has revealed its good performance in large dataset multi-label classification applications as well as in regression applications [26,27]. It is a generalization of a Single hidden Layer Feedforward Network (SLFN) [28,29,30,31,32,33]. To give a brief description of the essence of the ELM algorithm, we use the same notation as [27], who provides a descriptive figure with the same symbols. Consider the training data set that is formed by N samples with the format:

(5) $$\;\left\{x_{j},\;y_{j}\right\}\in \;{ℛ}^{d}\;x\;{ℛ}^{m},\;i=1,\;\ldots N\;$$

where *x_j_* is the input vector of sample *j^th^*, *y_j_* is the corresponding class of *x_j_*. The single hidden layer of ELM is composed by L nodes, each one has an activation function G (*a_j_*, *b_j_*, *x*) where *a_j_* is the associated connection weight vector and *b_j_* is the bias. The parameters *a_j_* and *b_j_* are randomly assigned. The hidden layer output matrix *H* is defined as:

(6) $$\;H=\left[\begin{array}{c} h\left(x_{1}\right)\\ \vdots \\ h\left(x_{N}\right) \end{array}\right]={\left[\begin{array}{ccc} G\left(a_{1},b_{1},x_{1}\;\right) & \ldots & G\left(a_{L},b_{L},x_{1}\;\right)\\ \vdots & \ddots & \vdots \\ G\left(a_{1},b_{1},x_{N}\;\right) & \ldots & G\left(a_{L},b_{L},x_{N}\;\right) \end{array}\right]}_{NxL}\;$$

The algorithm must compute the output weight vector *β* as:

(7) $$\;\beta =H^{†}Y\;$$

where *H^†^* stands for the Moore-Penrose inverse of matrix H and Y correspond to the class labels of the training data set:

(8) $$\;Y={\left[\begin{array}{c} y_{1}\\ \vdots \\ y_{N} \end{array}\right]}_{Nxm}$$

The output of the ELM is the optimal weight *β˜* of the network, where *β˜* is the least-squares solution of *β*.

The fitness function is applied to an individual by calculating through the ELM the correct classification rate of each of the groups of its grouping part on the training dataset. The best rate is assigned as a fitness value to the individual and the classification rates of the rest of the groups are discarded. The best classification rate determines the features selection that classifies the individual with the best accuracy among all the groups in its grouping part. The features selection is identified by the group with the best classification rate of the grouping part. The rest of the groups have no interest for the solution.

### 3.4. Statistical Analysis

As mentioned before, the spectral data sets have 1310 values along the frequency range 20 Hz–22.05 kHz. The classification by 1310 characteristics is far from being achievable. The objective of the GGA is to slash the number of features that are capable to classify samples within any composition of Table 1. The algorithm must solve a wrapper feature selection [34] where the GGA minimizes the output regressor. The solution is characterized by unknown group of features in size and composition. The reduced set of characteristics determined by the GGA among the 1310 must be able to classify any spectrum of the evaluation data set (unknown by the training data set) according to the types defined in Table 1. We want to select a limited number of attributes, around 40 or 50 features, which are able to perform the classification not worse than 90% successful.

The training, testing, and validation sets are disjoint sets formed from the 552 data. The training set is generally made up of 80% of the total data, the test data set contains between 10% and 15% and the evaluation set consists of the rest of the data (5–10%). We have performed the experiments with training, test and evaluation data set sizes of 80%, 15%, and 5%, respectively. The GGA trains first with a more numerous set, and then it tests with the testing set getting solutions for the selection of a set of attributes. The final solution is applied in the validation set in order to evaluate the accuracy of the solution provided as classifying criteria.

The population size of the GGA is fixed to N_ind_ = 50 individuals. The selection procedure of the GGA is based on tournament selection. The tournament selection operator has provided very good results in previous applications [35,36], and its implementation is quite easy. An offspring population of size 0.5 N_ind_ is obtained by applying crossover and mutation operators over the N_ind_ ones. The round of tournament is carried out with the merged population that is formed by parents and offspring, based on the best fitness values of the fighters. The foe is chosen at random for every tournament. The N_ind_ individuals with more won tournaments survive for the next generation.

The algorithm runs until any of two stopping conditions holds: convergence of the population or maximum number of generations *G_max_* has been reached. The convergence is estimated by comparing the fitness of the best solution in the population *f*_best_ with the average fitness of the population *f_average_*. We use a value of epsilon = 0.001 to carry out the comparison of the following equation:

(9) $$\;f_{average}-\;\epsilon \;\leq \;f_{best}\;\leq \;f_{average}+\;\epsilon \;$$

The maximum number of generations *G_max_* must be set in such a way that the algorithm is able to find out quality solutions, within a reasonable computation time. In our work, we have established *G_max_* = 100.

## 4. Results and Discussion

### 4.1. Acoustic Response Spectrum

We applied the GGA on 552 measurements of acoustic response spectra corresponding to 23 different types of samples (see Table 1). A detailed observation of them reveals that each spectrum has particular marks that are associated to the chemical composition of the sample, but it is extremely difficult to identify a set of frequencies for a proper classification, due to the large number of spectral lines. As an example, we selected the samples 2, 11 and 14 which with very similar composition in pairs:

- Sample 2: 6% ethanol by volume and 0 g/L of fructose
- Sample 11: 6% ethanol by volume and 2 g/L of fructose
- Sample 14: 10% ethanol by volume and 2 g/L of fructose

**Figure 5:**
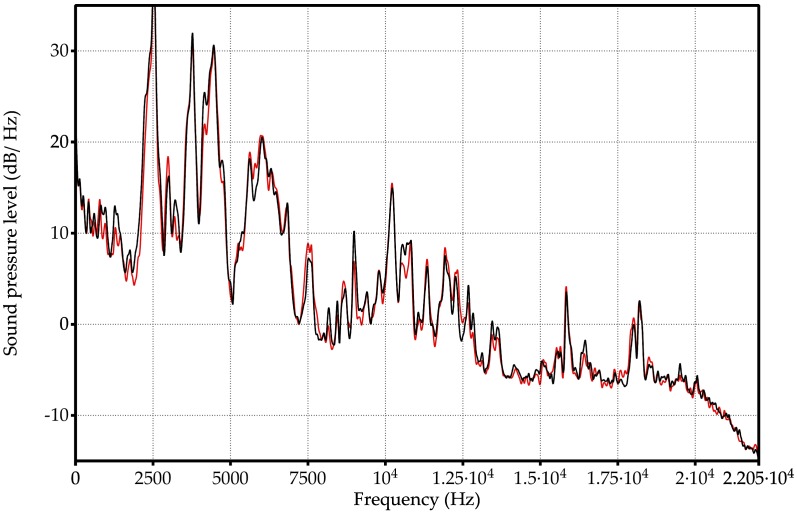
Spectral information of the vibrational absorption bands of two liquid samples with 6% ethanol by volume: sample number 2 (black line) and sample number 11 (red line). The hand classification according the sound pressure level is a hard task for two types of the Table 1 because the lines black and red are very close. It is unapproachable for 23 different types.

**Figure 6:**
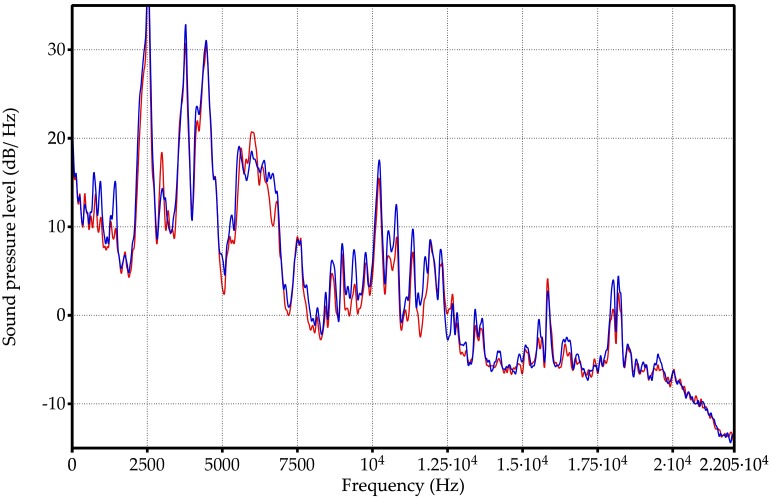
Spectral information of the vibrational absorption bands of two liquid samples with similar composition, both with 2 g/L of fructose: sample number 11 with a red line and sample number 14 (blue line). Both lines are so close that they make it difficult to classify them according of the frequency spectrum.

The Figure 5 shows the spectra of two measurements corresponding to samples 2 and 11. The sample 2 is defined with the black line whereas the sample 11 is plotted with red color. The spectra are defined by the sound pressure level (dB/Hz) as a function of the audible frequency range. Both of the curves are very close because their compositions are similar (same concentration of ethanol but different quantity of fructose). Observing the figure, the difficulty to select by hand a set of frequencies as a classification criterion between two different compositions is evident. The selection of a reduced set of frequencies as classifier for the 23 types in Table 1 is much more complex. Similar observation comes from the Figure 6, which compares two spectra of measurements of samples 11 (red line) and 14 (blue line). The spectra are very similar due to both samples have the same quantity of fructose, but different concentration of ethanol. However, the spectra are different besides being very complex to identify a particular sample through only their spectra.

The objective of this work consists of defining a reduced group of frequencies that is capable of classifying the 23 samples. To show the difficulty in determining a collection of frequencies as criteria for classification we have highlighted the frequencies selected in our experiments on Figure 5 and Figure 6. The selected frequencies group is able to classify the 23 types of samples. The size of the group is 36 frequencies and they are registered in Table 2. Note that the 36 frequencies are not needed to differentiate just two samples in any of the previous figures. Also, it is not the best group to classify just two types of chemical compositions because the whole group has been formed as 23 types classifier.

### 4.2. Feature Extraction

The GGA was executed to get a frequency group suitable to classify the 23 types of samples. The algorithm was executed about 20 independent simulations in a processor 2.7 GHz Intel Core i7. Each simulation took near 5 h (290 min). The values of the specific parameters of the GGA and ELM used as fitness function are the following:

Population size *N_ind_* = 50 individuals
Mutation probability = 0.1
*G_max_* = 100
Training data size = 80%
Testing data size = 15%
Validation data size = 5%
Number of neurons of ELM = 23

### 4.3. Classification

The Table 2 collects the group of frequencies in the audible sound frequency range solved by the GGA as classification criterium for the 23 types of samples of Table 1. Table 2 contains 36 frequencies that were selected from the 1310 frequencies of the spectra. Due to there being an appreciable variation between the values of sound pressure level (dB/Hz) for the high and low frequencies of the table, we have divided the set of 36 frequencies into four subgroups: low frequencies, low-medium frequencies, medium-high frequencies, and high frequencies. Table 2 includes this color code to differentiate the four sets of frequencies: green for the 18 lowest frequencies in the table, yellow for the five low-medium frequencies, red for the next five high-medium frequencies, and purple for the eight highest frequencies of Table 2. When considering four subgroups we can compare similar values in the energy density spectrum and thus zoom in the following figures in the document.

It is very interesting that the lowest frequency selected by the automatic classifier is 286 Hz, near the resonance peak of the sense of touch in the human fingerprints, although no information about this was supplied to the algorithm.

In order to check the stability of the ELM with the frequencies set of Table 2, we have made a battery of 60 random and independent iterations on the validation data set, unknown data set for the ELM during training period. The correct classification average rate or the accuracy of the ELM was 95% and the value of square of the correlation coefficient was Rho^2^ = 0.94. These results let us consider the solution frequency group as a quality classifier for the 23 types of samples. The Table 3 shows the average value for the 20 first iterations, with very similar conclusions.

**Figure 7:**
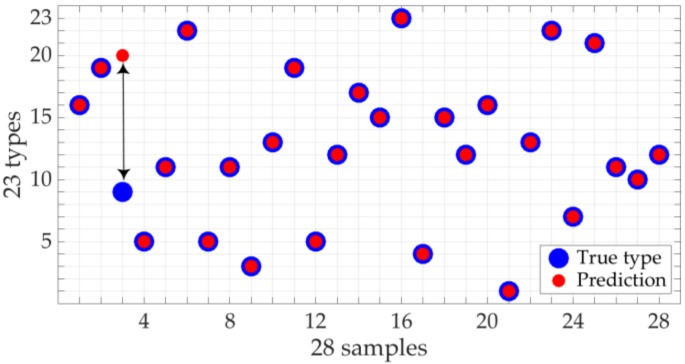
Extreme Learning Machine (ELM) classification of the validation data set in the first iteration of the Table 3. The x-axis corresponds to the measurements of the data set. The y-axis collects the 23 types of samples of the Table 1. Only one measurement is wrongly classified as type 9, whereas the right type is 20, marked with the black double arrow.

The Figure 7 shows the classification result of the first iteration of Table 3, which has an accuracy of 96% and a Rho^2^ value 0.89. The left part of the Figure 7 plots with a blue cross the prediction for each of the 28 measurements (5% of total data), whereas the red star indicates the actually type. The right part of the Figure 7 graphs the regression curve of the classification. Both plots mark only one case that is wrongly classified, which means the 96% accuracy mentioned before.

**Figure 8:**
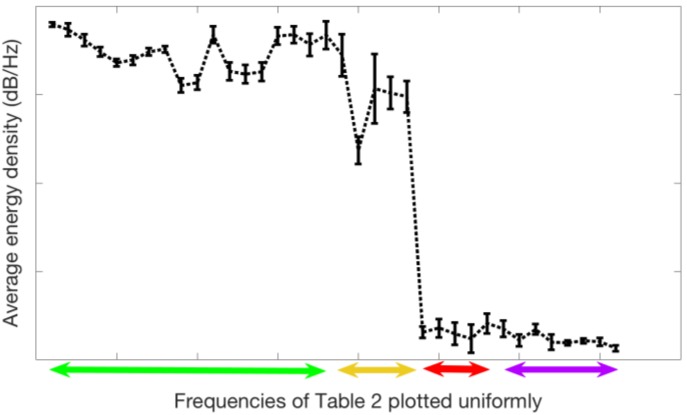
Average values and standard deviation of the spectrum of the all measurements of the type 11 in the whole data set. The x-axis includes the 36 selected frequencies of Table 2 plotted uniformly. The colors green, yellow, red, and purple correspond to the color in that Table.

We now analyze the quality as classifier of the 36 selected frequencies group of Table 2 according to the 23 types of samples of the Table 1. First, we are interested to obtain a spectrum pattern for the 23 types of sample. We constructed energy density patterns for each of the 23 types of samples from the average values of the energy density of the 24 experimental measurements taken from each of the types and in each of the 36 frequencies that were selected by the algorithm as a classification criterion. To evaluate the validity of the average values as a behavior pattern, the standard deviation is calculated for each frequency and for each type. The Figure 8 shows the average energy density values and standard deviation for the measurements in the total data corresponding to the sample 11 at the 36 selected frequencies. The average value of the standard deviation for all experimental measurements of type number 11 is less than 5%. A standard deviation of 5% is acceptable as a valid pattern, so we can take the average curve of energy density spectrum as a pattern for the sample 11. The similar process has been carried out for the rest of samples of Table 1.

Note that the frequencies are uniformly distributed on the X-axis. The Y-axis represents the average energy density of the spectra of the measurements in our experiments, which range is [−1, 1] dB/Hz. Note the energy density values differ widely for the 36 frequencies. Due to our need to compare similar values between spectral patterns, the frequencies of Table 2 is divided in four subgroups as mentioned before regarding the color code in the table. The Figure 8 is tagged on the X-axis the labels: low, low-medium, high-medium, and high frequencies groups.

### 4.4. Discussion

**Figure 9:**
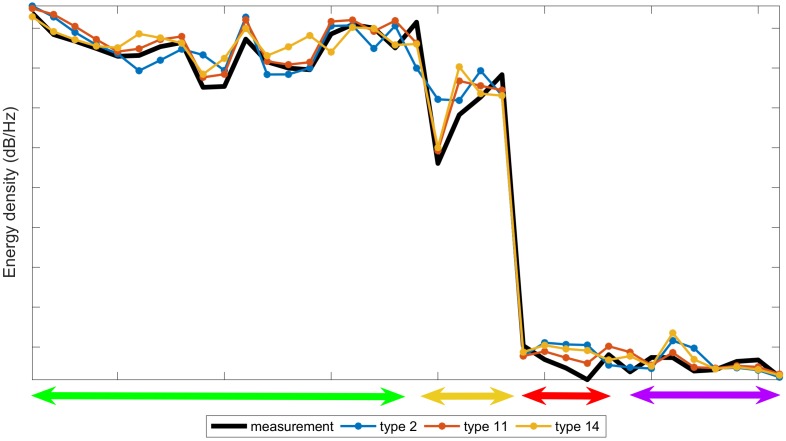
Comparison of the spectra data of a measurement of type 11 and the spectra data average of types 2, 11 and 14. The x-axis includes the 36 selected frequencies of Table 2 plotted uniformly. The colors green, yellow, red, and purple correspond to the color in that Table.

The Figure 9 plots the curves of spectral data of the 36 selected frequencies by the GGA for an individual measurement of type 11 (wider black line in the figure) and the average spectral information of types 2 (blue line), 11 (red line), and 14 (yellow line). We selected types 2 and 14, because they have very similar composition to type 11, so the patterns are also similar: type 2 with 6% ethanol by volume and 0 g/L of fructose, type 11 with 6% ethanol by volume and 2 g/L of fructose, and type 14 with 10% ethanol by volume and 2 g/L of fructose. Note that the black line (measurement of type 11) is more alike to the red one (pattern of type 11) than the others, especially in the two medium groups of frequencies. When we compare types with non-approximate compositions, the differences in the graph are more relevant. The most difficult task consists of comparing patterns with very close compositions.

**Figure 10:**
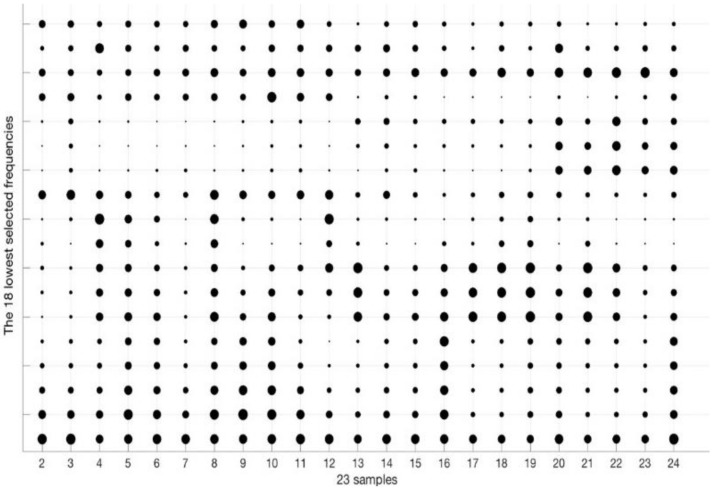
Bubbles graph with the average spectra information of the 23 patterns for the 18 lowest frequencies (green color) of the Table 2. The larger bubble corresponds to an energy density of 0.96746 dB/Hz. The smaller circle is associated with an energy density of 0.45294 dB/Hz.

To make easier the visual comparison of patterns, we use a bubbles graphical representation for the energy density values, where the value of energy density is proportional to the radius of its bubble. Bubble graphs allow for comparing the spectral data of a particular measurement easier than Figure 9. The Figure 10 shows the spectral fingerprint of the 23 liquid samples of Table 1 for the first subgroup of frequencies (the 18 lowest one). Each vertical line corresponds to a type of sample. The *Y*-axis refers to the frequencies that are distributed in Table 2. The radius of each bubble is proportional to the average energy density value of each frequency of the spectrum. In this figure, the maximum radius corresponds to an energy density value 0.96746 dB/Hz, whereas the minimum bubble depicts the value 0.45294 dB/Hz. Note that every type has its own mark, there are not two types with the same pattern. Thus, the classification process consists of the identification of patterns, finding the configuration more similar to a specific measurement.

**Figure 11:**
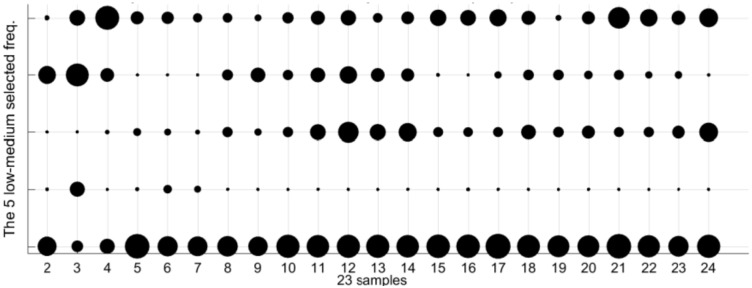
Bubble graph with the average spectra information for the 23 patterns for the 5 low-medium frequencies subgroup (yellow color in Table 2). The larger bubble in the graph corresponds to an energy density of 0.81514 dB/Hz. The smaller circle is associated with an energy density value of −0.061806 dB/Hz.

**Figure 12:**
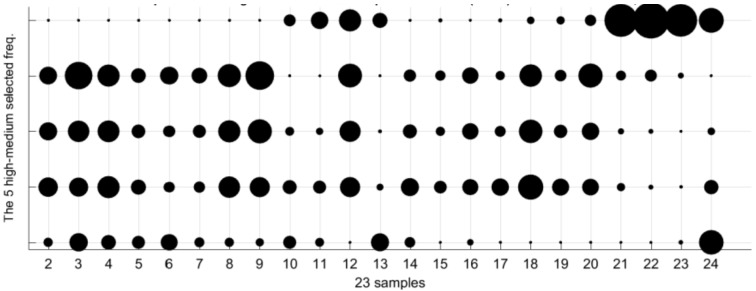
Bubble graph with the average spectra information for the 23 patterns for the 5 high-medium frequencies subgroup (red color in Table 2). The larger bubble in the graph corresponds to an energy density of −0.63674 dB/Hz. The smaller circle is associated with an energy density value of −0.92144 dB/Hz.

**Figure 13:**
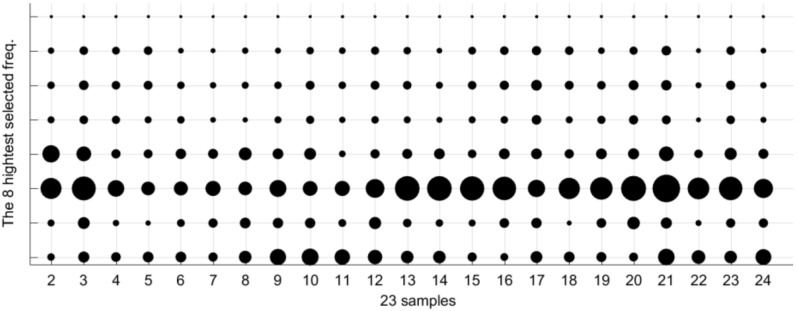
Bubble graph with the average spectra information for the 23 patterns at the eight highest frequencies selected by the algorithm (purple color in Table 2). The larger bubble corresponds to an energy density value of −0.69914 dB/Hz, whereas the smaller circle is associated to an energy density value of −0.96168 dB/Hz.

We can appreciate some types with similar but different patterns, for example, the type 2 (0% ethanol and 6 g/L of fructose) and type 3 (0% ethanol and 10 g/L of fructose). The spectral information of the 23 patterns according the other three frequencies subgroups is plotted in Figure 11, Figure 12 and Figure 13. As in the Figure 10, the three of them also present differences between very similar types. Each graph has particular bubble size in order to adjust the biggest bubble radius to the highest energy density value (dB/Hz) in each figure. In the Figure 11 (low-medium frequencies group), the maximum value of the energy density is 0.81514 dB/Hz and the minimum value is 0.061806 dB/Hz. Figure 12 shows the average energy density values of the 23 patterns for the high-medium frequencies are in the interval [−0.63674–0.92144] dB/Hz. The Figure 13 shows the energy density values for the 8 highest frequencies of Table 2 in the value range [−0.69914, 0.96168] dB/Hz.

Fructose increases the viscosity of the water solution, producing a higher sound attenuation in the audible frequency range. This could explain some of the changes that were seen in the acoustic spectra. However, a complete theoretical model that is able to reproduce the fine details of the experimental results would be very difficult. The effect of ethanol is even more complex, and the model should be considered at a molecular level to reproduce the obtained spectral data.

Most of the published works use infrared spectroscopy to study sugar-water binary solutions. Acoustical attenuation spectra of some carbohydrate aqueous solutions have been found only in [37], but in the range 12 kHz–2 GHz. The author mentions relaxation terms reflecting conformational changes, but in the high frequency band. Attenuation and sound velocity changes at audible frequencies are explained in [38] by the combined effect of viscous and thermal losses.

Acoustic spectroscopy studies of ethanol-water binary solutions cannot be found in the literature, to our best knowledge, but [39] presented evidence of self-association of water and ethanol molecules by means of low-frequency Raman spectroscopy. This could explain some properties of the measured acoustic spectra in this work, but there is not a complete theoretical model to confirm it.

**Figure 14:**
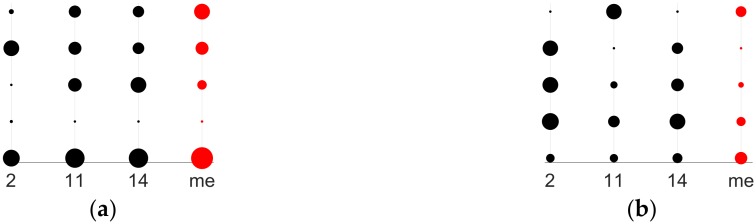
Bubble charts comparing a measurement of type 11 (bubbles of red color) with the patterns of composition more similar (color black for the patterns of types 2, 11, and 14). We included only the low-medium (**a**) and medium-high (**b**) frequency groups of Table 2 because the differences are more marked than with the rest of the frequencies. The measurement is correctly classified because it is more similar to the type 11 pattern in these frequencies. With the rest of the patterns, the differences are greater, and those patterns are discarded.

**Figure 15:**
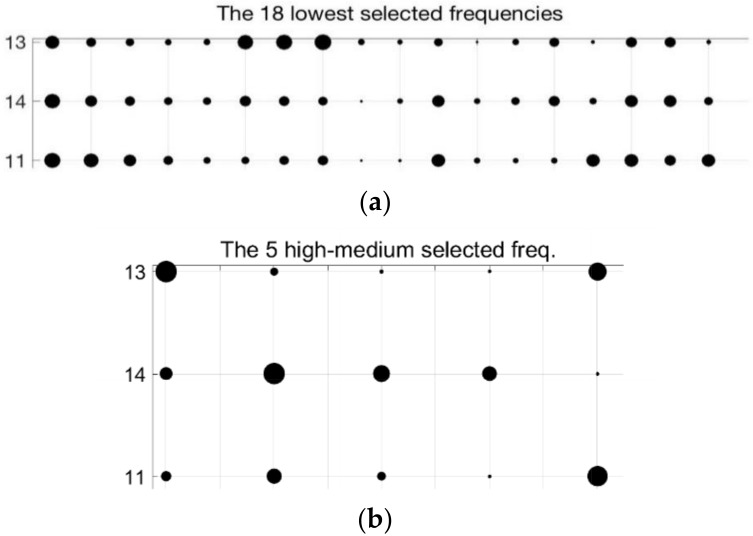
Bubbles graphs of types 11, 13, and 14 for some frequencies of Table 2. These sets of frequencies make it possible to differentiate patterns 11, 13, and 14, even though they have a similar composition in fructose and ethanol. (**a**) The 18 lowest frequencies. The bubble with the maximum radius corresponds to 0.9361 dB/Hz, the minimum radius in the graph is for the 0.5531 dB/Hz; (**b**) The 5 high-medium frequencies, where the maximum radius corresponds to −0.7910 dB/Hz, the minimum radius in the graph is for the −0.8882 dB/Hz.

The Figure 14 extracts some information of Figure 9 to make the classification clearer. The figure graphs the 10 medium groups of frequencies because the differences between the bubble sizes of the three types (patterns 2, 11, and 14) are more evident. The bubbles fingerprints of the three ones are in black color and compared to the bubbles line of a particular measurement of type 11 in red color. The comparison of the vertical bubbles lines in both graphs of Figure 14 discard the type 2 as candidate for the classification of the measurement. The bellow graph of the figure lets us correctly classify the measurement thank to the differences of vertical bubbles lines of types 11 and 14. Figure 15a corresponds to the 5 low-medium frequencies group, where the maximum radius is 0.8409 dB/Hz, whereas the minimum radius in the graph is for the 0.0835 dB/Hz. Graph in the right is associated to the 5 high-medium frequencies group. The bubble with the maximum radius is −0.7785 dB/Hz, the minimum radius in the graph is for the −0.9594 dB/Hz.

The 36 frequencies that were selected by the optimization GGA in Table 2 is one of the best to classify the total 23 types of Table 1. It does not mean that all frequencies contribute in the same way in the classification process. To appreciate this idea, we consider in Figure 15 the bubbles lines for the types 11 (6% ethanol by volume and 2 g/L of fructose), 14 (10% ethanol by volume and 2 g/L of fructose), and 13 (10% ethanol by volume and 1 g/L of fructose) for the frequencies groups where the lines are more different. Other frequencies help to distinguish other groups. The lines are plotted horizontally in order to reduce the used area on paper.

It can be seen that the results show that this new technique is able to detect and classify several water, ethanol, and fructose dissolutions with a precision of 0–13% by volume of ethanol and 1–3 g/L of fructose in water. The correct classification average rate that was obtained by the grouping genetic algorithm was 96%. Thus, this new method can detect up to 1 g/L of fructose and 1% ethanol by volume in liquid samples with an error lower than 10%.

## 5. Conclusions

We have described a new haptic sensor that is based on vibration to determine the chemical composition of liquid mixtures according to their composition of ethanol and fructose in a non-invasive way with minimal cost. The analyzed spectrum range is 20 Hz–22.05 kHz. The spectral information from the vibrational absorption bands of liquid samples is analyzed by a Grouping Genetic Algorithm. An Extreme Learning Machine implements the fitness function that is able to select a reduced set of frequencies from the acoustical response spectrum of the samples to resonant ticking sounds. It is enough to analyze the response of the spectrum at a few frequencies instead of studying the whole spectrum.

The experiments have been performed with 552 measurements belonging to 23 samples of water, ethanol, and fructose mixtures (50 mL) of distilled water with a concentration of ethanol of 0%–13% by volume and fructose 0–3 g/L. The work concluded that less than 40 frequencies are enough to process the characterization with accuracy greater than 80%. After 20 iterations, the average accuracy of the operation is 96%, any of them with accuracy better than 82%. The method can differentiate up to 1 g/L of fructose and 1% ethanol by volume in liquids samples with an error lower than 10%.

The optimization algorithm provides several solutions with similar quality. The solution is not unique. Also, it is important to note that if the number of types is modified, the set of selected frequencies in the solutions will be also changed. In other words, each frequency has a different contribution to the characterization result. Future research will be orientated to analyze other chemicals substances, more complex mixtures, and to improve the accuracy of the sensor.

## Figures and Tables

**Table 1:** Numerical labels from 2 to 24 for the experimental samples simulating different types of alcoholic beverages. The labels classify the samples according to their concentrations of ethanol and fructose.

Id Sample	2	3	4	5	6	7	8	9	10	11	12	13
Ethanol (%)	6	10	11	12	13	0	0	0	6	6	6	10
Fructose (g/L)	0	0	0	0	0	1	2	3	1	2	3	1
**Id Sample**	**14**	**15**	**16**	**17**	**18**	**19**	**20**	**21**	**22**	**23**	**24**	
Ethanol (%)	10	10	11	11	11	12	12	12	13	13	13	
Fructose (g/L)	2	3	1	2	3	1	2	3	1	2	3	

**Table 2:** Set of 36 frequencies offered by the GGA as classification criterion among the 23 types in Table 1. The classification process considers the sound pressure values (dB/Hz) at these frequencies. Because these values are very different for the low frequencies and for the high ones, the following figures analyze separately the sound pressure levels at the lowest frequencies (green color in the table), the low-intermediate ones (yellow color), the intermediate-high (red color), and the highest frequencies (purple in the table). The colored arrows are also represented in the Figure 8.

Low Frequency 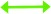	Id	1	2	3	4	5	6	7	8	9
	Frequency (Hz)	286	622	639	656	673	774	791	807	875
	Id	10	11	12	13	14	15	16	17	18
	Frequency (Hz)	976	2,658	3,095	3,112	3,129	3,348	5,619	5,753	6,881
Low-Medium 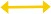	Id	19	20	21	22	23				
	Frequency (Hz)	7,469	9,623	10,430	11,843	12,045				
High-Medium 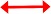	Id	24	25	26	27	28				
	Frequency (Hz)	16,150	16,268	16,284	16,301	16,739				
	Id	29	30	31	32	33	34	35	36	
High Frequency 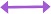	Frequency (Hz)	17,513	19,380	19599	19,750	20,928	20,944	20,961	22,021	

**Table 3:** Results of the correct classification rate of the validation data set for 20 independent iterations using the 36 frequencies in Table 2. The results are shown as the correct classification rate as accuracy and square of the correlation coefficient Rho.

Iteration	1	2	3	4	5	6	7	8	9	10
Accuracy	0.96	1.00	1.00	0.86	0.96	0.93	0.93	0.96	0.89	0.93
Rho^2^	0.89	1.00	1.00	0.94	0.99	0.91	0.94	1.00	0.92	0.97
**Iteration**	**11**	**12**	**13**	**14**	**15**	**16**	**17**	**18**	**19**	**20**
Accuracy	0.96	1.00	0.82	1.00	0.96	0.93	0.89	0.93	0.86	0.96
Rho^2^	1.00	1.00	0.89	1.00	1.00	0.97	0.97	0.92	0.87	1.00
